# Sexual Coach in High-Functioning Autism: A Growing Need

**DOI:** 10.3390/brainsci12080964

**Published:** 2022-07-22

**Authors:** Rocco Salvatore Calabrò, Giovanni Pioggia, Marianna Contrada, Antonio Cerasa

**Affiliations:** 1 IRCCS Centro Neurolesi “Bonino Pulejo”, 98164 Messina, Italy; 2 Institute for Biomedical Research and Innovation (IRIB), National Research Council of Italy, 98164 Messina, Italy; giovanni.pioggia@irib.cnr.it (G.P.); antonio.cerasa76@gmail.com (A.C.); 3 S. Anna Institute, 88900 Crotone, Italy; marianna.contrada@gmail.com; 4 Pharmacotechnology Documentation and Transfer Unit, Preclinical and Translational Pharmacology, Department of Pharmacy, Health Science and Nutrition, University of Calabria, 87036 Rende, Italy

When individuals with autism spectrum disorders (ASD) reach adulthood, they may experience a set of challenges related to sexual health, dating and romantic relationships. Many people, including parents and caregivers, still hold stereotypes about the sexuality of this population, as they have the perception that people with ASD are asexual and not interested in sex. Indeed, they are almost seen as “eternal children” [1,2].

Due to the main symptoms of ASD (deficits in social and interpersonal skills) as well as to the lack of sex education, some autistic individuals may develop above-average or non-normative sexual behaviors [3]. Generally, no differences between autistic individuals and the normo-typical population have been found in seeking sexual and romantic relationships [4]. Specifically, several studies suggest that autistic males, unlike females, engage more in solitary sexual activities and have greater desire and are more romantic [5]. However, some problematic sexual attitudes have been reported, such as hypersexual and paraphilic behaviors [6]. In the first case, the disorder is characterized by repetitive and persistent sexual urges that are experienced as uncontrollable. In a study by Schöttle and colleagues [6], hypersexual behaviors were present in 30.4% of males with ASD compared with 10% of females. Moreover, the same authors showed that paraphilias were more frequent in ASD males with cognitive impairment and included excessive masturbation, exhibitionist behaviors, pedophilic fantasies, fetishistic fantasies, sadomasochistic behaviors or other forms. Female patients with ASD indeed showed no differences in the frequency of paraphilic fantasies or behaviors compared with healthy subjects (healthy control, HC), except in the frequency of masochistic behaviors [6].

Nonetheless, patients with ASD, especially those with high-functioning autism, have the right to have the best possible quality of life, including sexual well-being. This is achievable only if this “hot” but fundamental topic is discussed with both the patients and their parents to overcome prejudice and false myths. To this end, a recent review found a growing awareness of the desire for sexual and intimate relationships in individuals with ASD [1]. Unfortunately, the core impairments of ASD lead to difficulties in establishing the knowledge and skills required to reach a healthy sexuality and facilitate relationships [1]. However, there has been little attention paid to the need for sexual education for youth with ASD during this transitional period between adolescence and adulthood [2]. The existing research focuses mainly on prevention of sexual abuse of people with intellectual disabilities, including ASD, but attention should be paid to other aspects of sexuality. Indeed, it has been shown that young adults and adolescents with ASD know much less about sex than peers without disabilities. As a consequence, a lack of sex education makes this population more vulnerable to unplanned pregnancy and sexually transmitted disease, and they are more likely to be sexually abused.

To properly deal with this important topic and to better support these adolescents, all healthcare professionals and persons involved in ASD care should be trained in human sexuality [7,8], and sexual education should be provided to all individuals with high-functioning ASD. In fact, growing evidence demonstrates that parents of people with ASD declared having little support to educate their offspring and provided less sex education to children with intellectual disability and severe symptoms. Accordingly, psychosexual education programs that are tailored to suit developmental and cognitive differences of ASD individuals are needed in order to increase knowledge and improve parent–child communication, especially for younger and high-functioning adolescents [9,10].

In addition to sex education with specific training for both patients and their caregivers, people with ASD may benefit from sexual coaching. A sex coach is a trained professional who helps people with sexual, intimacy and relationship issues, including sexless marriage, low libido and sexual dysfunction, but also guides their clients to fully grasp their sexual potential through education, training and communication.

For anyone who has experienced changes resulting from a disability and does not have a partner to work with, the coach or surrogate can help explore and develop sexual potential. Such coach partners differ from prostitutes in that they are professionally trained and work in a therapeutic situation comprising the client, the coach and the licensed supervising therapist. A coach usually feeds information back to both the client and the therapist, so that together, the surrogate, the client and the therapist work in a sort of therapeutic “love triangle”. During talk coaching sessions, the sex coach asks the therapist about the patients’ challenges and goals and then gives suggestions for improvement, as well as book and video recommendations.

During experiential sex coaching, the coach teaches how to be a better lover through talking as well as hands-on practices, including breath, touch, how to emit and share sexual energy, and how to verbally seduce and give pleasure to a partner. Therefore, sexual coaching may be really helpful to overcome sexual dysfunction in people with different neurological and psychiatric disabilities. Nonetheless, if, usually, patients with brain or spinal cord lesions often retain the capacity to consent to “sex activities”, this is not the case for individuals with intellectual disabilities, including ASD [11]. Indeed, if, on the one hand, patients with ASD have the right to live life to the fullest, pediatricians have practical concerns regarding their ability to consent to sex as well as avoid coercion and manipulation in sexual encounters [12].

To overcome this issue, the available educational training on sexuality and relationship requires a more thorough evaluation, both in terms of its efficacy in improving knowledge about sexual behaviors and of its impact on the capacity to make sexuality-related decisions [13]. In our opinion, this is possible only in people with high-functioning autism, as they somehow preserve or can potentiate this capacity.

In the future, an alternative solution for overcoming the mental and social taboos related to this topic could be the employment of a mediator of behavioral intervention. Social robots have become reliable tools for improving clinical outcomes, assisting therapists in teaching new skills or special educational needs to ASD individuals [14]. In sexual education, the employment of social robots has never been performed, although some industries are starting to market a new generation of sex robots. In the last few years, there has been a proliferation of sex robots into our lives, raising heavy controversy among the activists, therapists and scholars from various disciplines [15]. Sex robots are realistic mannequins with variable ages, appearances and textures, and customizable sexual organs. The employment of these sexual anthropomorphic devices in mental health has been proposed as “harm limitation” for reducing sexual crimes [14], although this hypothesis has been largely rejected. Cox-George and Bewley [14] claimed that without evidence-based support for the application of sex robots in healthcare, their employment is specious. It could be plausible to hypothesize that sex robots will be helpful for patients who would benefit from sexual practice without pressure, although there are several conditions that should be defined before applying these devices in individuals with ASD: (a) the degree and type of sexual dysfunctions; (b) sexual orientation; (c) the degree of mental disability (i.e., socio-emotional skills); and, last but not least, (d) the type of psychosexual educational approach. After establishing these critical points, future clinical trials could be proposed for evaluating the real impact of these sex robots on sexual coaching and how these could impact well-being and mental health.
